# Salivary exosomal PSMA7: a promising biomarker of inflammatory bowel disease

**DOI:** 10.1007/s13238-017-0413-7

**Published:** 2017-05-18

**Authors:** Xiaowen Zheng, Feng Chen, Qian Zhang, Yulan Liu, Peng You, Shan Sun, Jiuxiang Lin, Ning Chen

**Affiliations:** 1 The Department of Orthodontics, Peking University School and Hospital of Stomatology, Beijing, 100081 China; 2 Central Laboratory, Peking University School and Hospital of Stomatology, Beijing, 100081 China; 3 The Department of Gastroenterology, Peking University People’s Hospital, Beijing, 100044 China; 4 The School of Life Sciences, Tsinghua University, Beijing, 100084 China

**Keywords:** exosomes, inflammatory bowel disease, proteomics, PSMA7

## Abstract

Inflammatory bowel disease (IBD) is an intestinal immune-dysfunctional disease worldwide whose prevalence increasing in Asia including China. It is a chronic disease of the gastrointestinal tract with unknown cause. Exosomes are small vesicles in various body fluids. They have diameters of 40–120 nm, and one of their functions is long-distance transfer of various substances. In this study, we investigated the contents of salivary exosomes in patients with IBD and in healthy controls to explore a new biomarker in patients with IBD. In this study, whole saliva was obtained from patients with IBD (ulcerative colitis (UC), *n* = 37; Crohn’s disease (CD), *n* = 11) and apparently healthy individuals (HC, *n* = 10). Salivary exosomes were extracted from samples, and the proteins within the exosomes were identified by liquid chromatograph-mass spectrometer (LC-MS/MS). The results showed that more than 2000 proteins were detected in salivary exosomes from patients with IBD. Through gene ontology analysis, we found that proteasome subunit alpha type 7 (PSMA7) showed especially marked differences between patients with IBD and the healthy controls, in that its expression level was much higher in the CD and UC groups. This exosomal protein is related to proteasome activity and inflammatory responses. So we conclude that in this research, salivary exosomal PSMA7 was present at high levels in salivary exosomes from subjects with IBD. It can be a very promising biomarker to release the patients from the pain of colonoscopy.

**Electronic supplementary material:**

The online version of this article (doi:10.1007/s13238-017-0413-7) contains supplementary material, which is available to authorized users.

## INTRODUCTION

Inflammatory bowel disease (IBD) includes ulcerative colitis (UC) and Crohn’s disease (CD). It is an immune-dysfunctional disease worldwide whose prevalence increasing in Asia including China. It is a chronic disease of the gastrointestinal tract of unknown cause. Previous reports have suggested that IBD may be related to gut microbes and/or immune regulatory mechanisms; more recent research shows that it may also be related to oral cavity (Rautava et al., 2015).

Recently research on IBD has focused on exosomes, spherical vesicles with a diameter of 40–120 nm (Vlassov et al., 2012). They contain diverse contents, such as proteins, including enzymes (Simpson et al., 2009). Exosomes also carry lipids (Siravegna et al., 2017) and, frequently, high levels of RNAs, among which miRNAs represent a considerable proportion (Bach et al., 2017). Exosomes are now considered to have multiple functions, including immune regulation, distribution of cell substances, and propagation of prion proteins and retroviruses (Ritchie et al., 2013). They also provide an important tool in drug delivery, using mesenchymal stem cells to secrete them (Lai et al., 2013). Exosomes are regarded as a major player in material communication between cells because of their ability to transport substances. In 2013, the Noble Prize for Physiology and Medicine was awarded to three scientists who determined the mechanism of exosome-mediated transport between cells. Previously, it was suggested that exosomes are messengers that transport substances between cells, but the mechanism was not understood. What’s more, exosomes have the ability of stability which help them move far away and not to rupture. These characteristics suggest that exosomes have potential use in clinical diagnosis and treatment (Zheng et al., 2014).

Exosomes exist in almost every type of cell and body fluid (Yamada et al., 2012). They also exist in saliva. As an important aspect of the oral cavity, saliva connects the mouth with the gastrointestinal tract and other body tissues and organs. Many oral diseases, such as ulcers, tumors, and many other lesions, exist not only in the mouth but elsewhere in the body. Similarly, the occurrence and development of systemic diseases can also influence changes in salivary biomarker expression and may even cause oral lesions. For example, Lau et al. (2013) suggested that pancreatic cancer and the oral cavity interact with each other via exosomes derived from the tumor. The oral cavity is frequently affected in patients by IBD. According to literature reports, the incidence of IBD with oral involvement is 0.5%–80% (Rowland et al., 2010). Oral lesions frequently co-occur with IBD intestinal damage and are often relieved when intestinal inflammation improves, suggesting that the occurrence of oral lesions is correlated with the incidence of IBD (Veloso, 2011). Thus, we suggest that oral saliva of IBD patients contains exosomes. Moreover, the contents in these exosomes may, at least partially, reflect the existence and development of IBD, and they may be great biomarkers of IBD due to the characteristics of exosomes.

A shotgun mass spectroscopy approach enables thousands of proteins to be identified at once; its efficiency and sensitivity render it a powerful technique in protein research (Dowell et al., 2008). As early as 2009, Rommel et al. (2009) used 1D-PAGE and LC-MS/MS to identify proteins in exosomes from colon carcinoma cells. In 2011, Choi et al. (2011) detected approximately 850 microvesicular proteins from ascites in patients with colorectal cancer. Those findings showed that intestinal cells secrete exosomes that may transfer between organs or influence the other organ’s exosomes. Thus, using a shotgun approach, deep and accurate exploration of proteins in exosomes is possible. Given this background, to explore differences in the salivary protein contents of exosomes between patients with IBD and healthy subjects, we used a shotgun proteomics approach to identify the protein composition of exosomes in various groups.

## RESULTS

### Electron microscopy images and membrane protein verification

**Figure 1:**
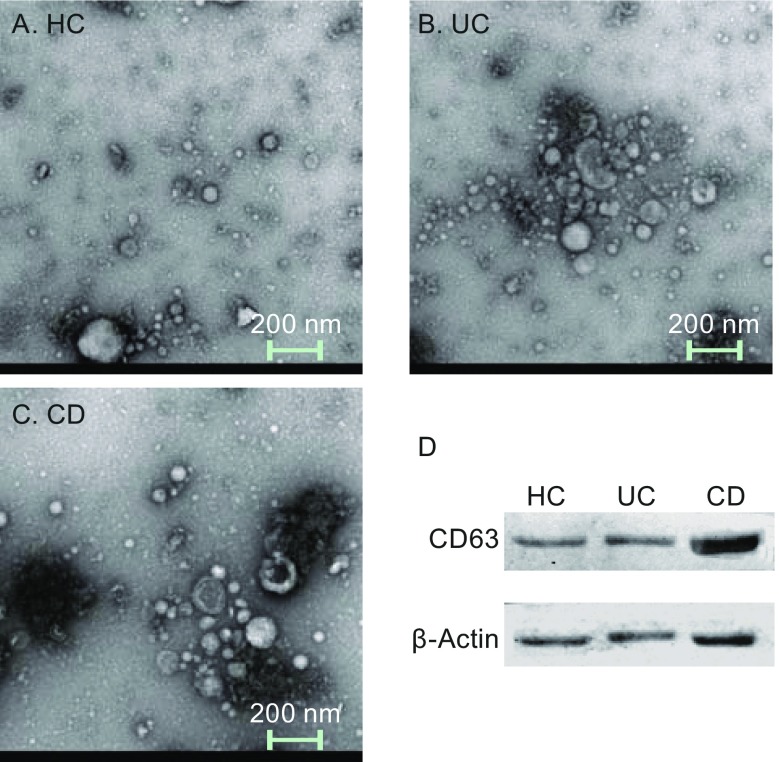
**Electron microscopy of exosomes**. (A–C) The images of salivary exosomes under electron microscopy of HC, UC, and CD patients. The scale bar is 200 nm and the exosomes are 30–120 nm in size and largely spherical in shape. The size of the exosomes did not differ markedly. (D) Western blotting of exosomes-specific CD63 and housekeeping gene β-actin. The existence of the vesicle membrane-protein confirms the extraction of exosomes

Figure 1A–C show intuitive images of salivary exosomes from patients with UC, CD and healthy controls. The scale bar is 200 nm; the exosomes are 40–120 nm in size and largely spherical in shape. Their bilayer lipid membrane can be seen. The size and shape of the exosomes did not vary markedly. These findings confirmed the presence of exosomes in the salivary samples. Western blotting of the exosome-specific CD63 and the housekeeping β-actin proteins as vesicle membrane-specific proteins was performed (Fig. 1D). Although the three groups exhibited some differences in expression profiles, all contained CD63 and β-actin, confirming the extraction of exosomes. The results of Fig. 1 are intended to illustrate that the method to extracting exosomes from the three sets of samples is stable, repeatable, and successful.

### Salivary exosomal proteins in healthy controls and patients with IBD

**Figure 2:**
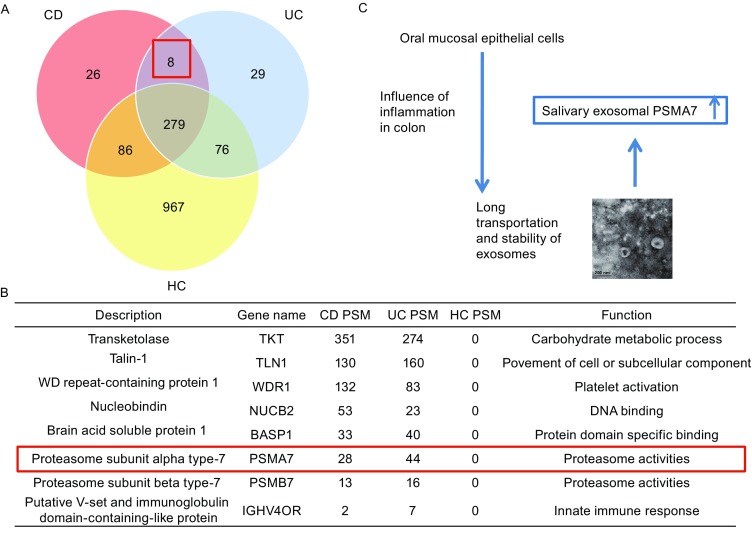
**Proteins in salivary exosomes of IBD and healthy groups**. (A) Comparison of the numbers of proteins in healthy controls and IBD patients. (B) 8 proteins that only exist in IBD patients (both CD and CD). The most reasonable protein that may interact between oral cavity and colon intestine is PSMA7. (C) PSMA7’s function in the inflammation or cancer in the digestive systems had been reported by many studies. The speculated way shows the expression of PSMA7 is high in the salivary exosomes of IBD patients. IBD may influence the oral mucosal cells to secret exosomes, which may express the similar protein pattern of colon tissues

**Figure 3:**
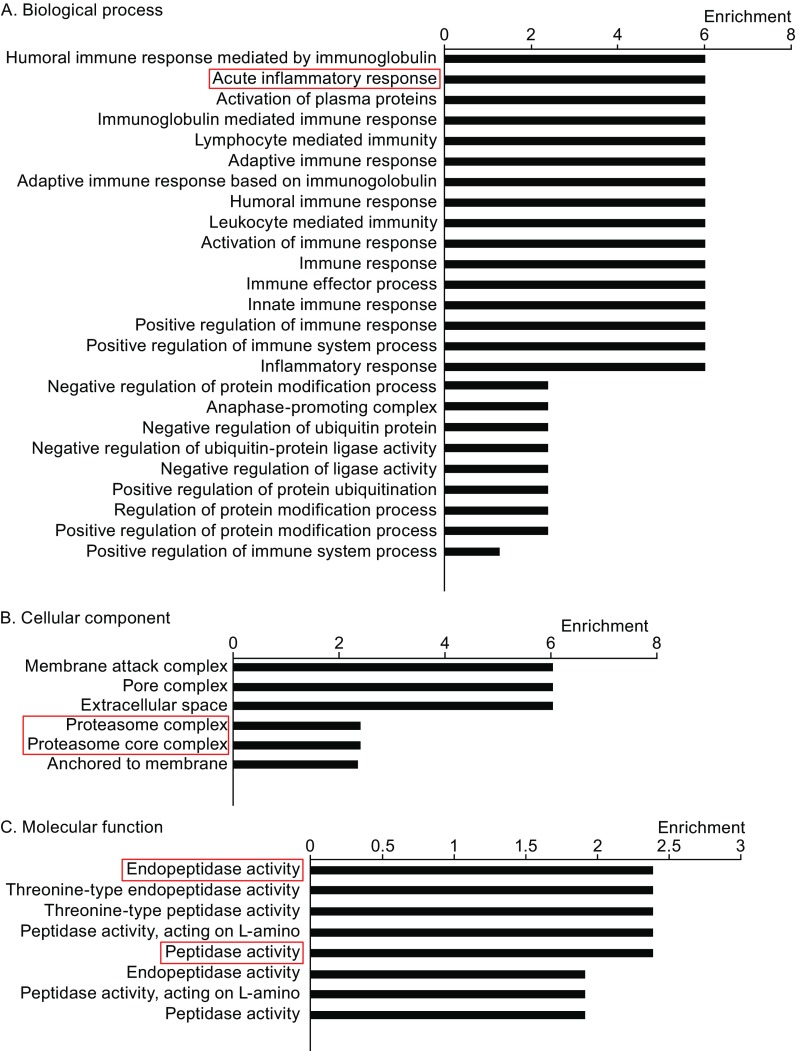
**GO analysis of 63 genes**. (A–C) GO analysis of the total 63 differentially expressed proteins was based on the shotgun proteomic results in the aspects of biological process, cellular component, and molecular function. Some highly enriched terms such as acute inflammatory response, proteasome complex, endopeptidase activity etc. were associated with inflammatory bowel disease

The protein profiles of salivary exosomes differed among patients with IBD and healthy controls. Raw data peptide-to-spectrum matches (PSM) were generated automatically from the system database and can provide a useful comparison dataset among the three groups of proteins, showing the expression level of some proteins (Zhang et al., 2014). Figure 2A is a venn chart and has three sets of samples. The intersection of the proteins (26 + 8 + 279 + 86 = 399) of all the CD samples is made into a red circle,which means these 399 kinds of proteins are exsisting in every CD individual sample. Similarly, there are 392 (8 + 29 + 76 + 279) kinds of proteins in every UC individual sample. The yellow circle is the collection of all the 1,408 (279 + 76 + 86 + 967) proteins in the healthy control group. It means the sum of all the proteins in each HC sample is 1,408. Every individual healthy sample may not contain all the 1,408 proteins. This screening ensures the 8 proteins are present in every IBD patient. 26 proteins are present only in every CD patient. 29 proteins are only present in every UC patient. So we chose eight proteins in the red box, which are present in all IBD patients and are not present in healthy patients. Details of the eight proteins are provided in Fig. 2B. In addition, 279 kinds of proteins in the middle of the three circles are present in all three sets of the samples. There may be examples of proteins that are reduced as well as elevated in the IBD groups in these 279 proteins. But compared to the specific 8 proteins only expressing in IBD groups, even if there are differences in the expression of the 279 proteins between IBD and healthy groups, their specificity may not be so good. So we are mostly concerned with the 8 specific proteins in the disease group. We provide all the protein number and description in each category in the Supplementary data 1. Next, GO analysis using the DAVID database was performed to analyze 63 (8 + 26 + 29) genes found only in patients with IBD in terms of biological processes, molecular functions, and cellular components (Fig. 3). Most GO terms with high enrichment score were related to acute inflammatory responses, proteasome complexes, peptidase activity, and immune responses.

### GO analysis of salivary exosomal proteins in patients with IBD

Shotgun mass spectra were used to identify proteins in salivary exosomes from patients with IBD (UC and CD groups). Using the same selection process to evaluate exosomal proteins in patients with IBD and healthy controls, 63 proteins that only express in the CD and UC groups were identified, and a GO analysis was performed. And those 63 proteins are distributed in a variety of GO terms concerning the molecular function, biological process, and cellular components (Fig. 3). GO analysis is conducted by the DAVID website. Data statistics are also derived directly from the DAVID database. The GO term we choose to study should have a high enrichment score and the *P* value of which should be lower than 0.05. Some GO terms with a high enrichment score, such as peptidase activity, acute inflammatory response, and membrane attack complex, were associated with inflammation and proteosomes. We are mostly concerned about the 8 proteins expressed only in the IBD group (both CD and UC). The 8 proteins distinguished the group of IBD disease and healthy controls. The proteins we choose should meet the following criteria: 1. The protein should be in the 8 proteins that only expressed in the IBD group; 2. The gene of the protein is in the GO terms with a high enrichment score. PSMA7 is distributed in the most GO terms with high enrichment score. Meanwhile a large number of literatures have reported that PSMA7 has increased in IBD intestinal fluid, tissue, or in colorectal cancer, or some other digestive diseases. In addition, PSMA7 is reported to be associated with intestinal inflammation and is associated with intestinal cancer metastasis. Thus, we focused on proteasome subunit alpha type 7 (PSMA7, NCBI GENE ID: 5688) within the peptidase activity group as a target protein.

### Verification of select specific proteins by Western blotting

**Figure 4:**
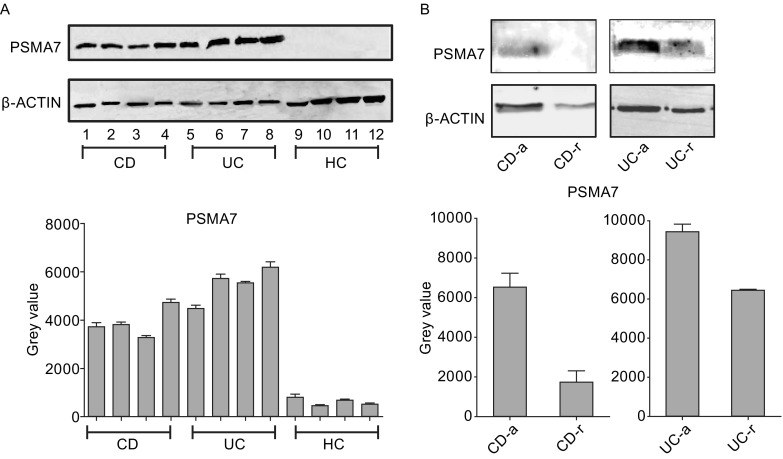
**Verification of PSMA7 in salivary exosomes**. (A) Salivary exosomal PSMA7 levels were detected by Western blotting among HC, CD, and UC groups. And PSMA7 exists mostly in CD and UC patients. (B) Salivary exosomal PSMA7 levels were detected in CD-active, CD-remission, UC-active, UC-remission patients. The result shows expression of PSMA7 reduced a lot in patients who are in the remission phase

Western blotting was performed to confirm PSMA7 expression in individual samples. The Western blotting results are shown in Fig. 4A. Levels of PSMA7 were significantly higher in patients with CD and UC compared with healthy controls, which showed negligible levels, confirming our previous results. Moreover, samples from patients at different stages were also evaluated by Western blotting (Fig. 4B). The results showed much lower exosomal PSMA7 levels in patients with UC and CD in remission than in patients with active diseases.

### Animal studies present that PSMA7 expressed the similar pattern in both colon and oral tissue

**Figure 5:**
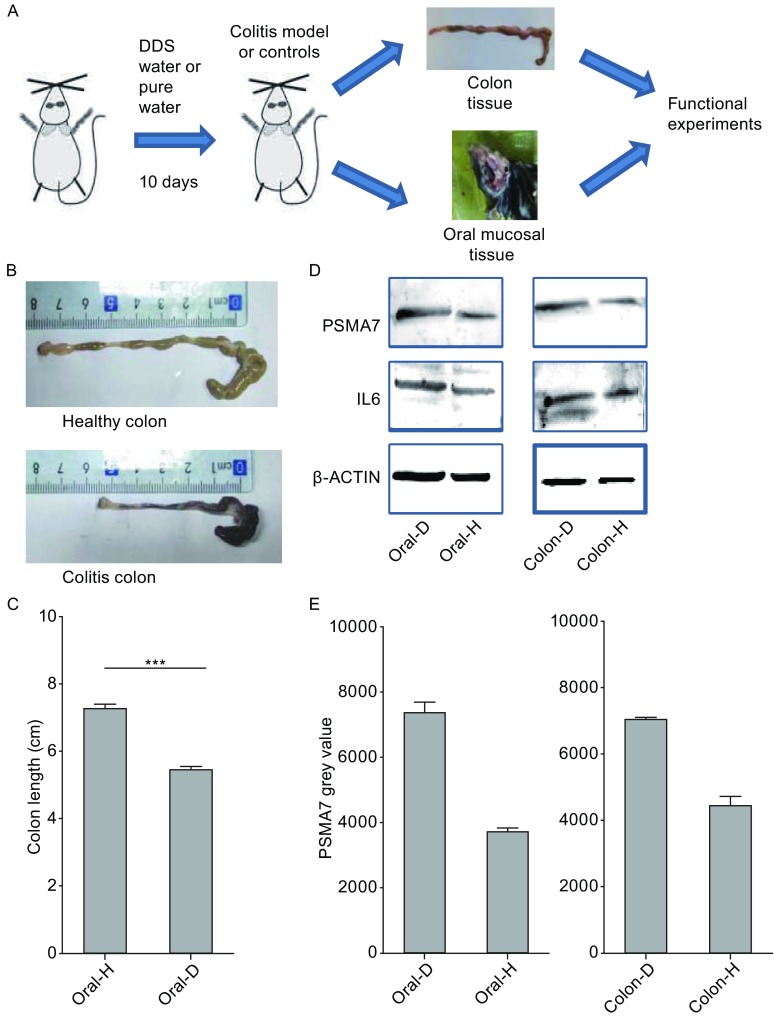
**Expression profile of PSMA7 in animal studies**. (A) Flow chart of the animal study. (B) Colon sections obtained from mice were analyzed for their outward appearance. (C) Colon length of the colitis model mice and control groups. (D) Proteins from the tissue of colon and oral cavity were extracted. The expression of PSMA7 is higher in IBD groups both in the colon and oral tissues

The model of IBD and healthy group was established with the male C57BL/6 mice. Figure 5A showed the flow chart of the animal study. Figure 5B showed the different phenotype of IBD mice colon and healthy mice colon. And Fig. 5C illustrates the different length of IBD colon and healthy colon. So the Fig. 5B and 5C showed the mice IBD model to be usable. Western blot showed the expression level of the protein in the oral and colon tissue from the IBD model and healthy controls. Higher expression of IL-6 in IBD colon supplements the effective model of IBD mice. Higher expression of PSMA7 in colon and oral tissue of IBD mice model may further illustrate that the expression pattern of salivary exosomal PSMA7.

## DISCUSSION

Studies of exosomes from patients have focused mostly on colorectal cancer. In contrast, little research has addressed salivary exosomes from patients with IBD. To our knowledge, our study is the first report on salivary exosomes in IBD.

### Extraction of exosomes from saliva

Exosomes are spherical vesicles, 40–120 nm in diameter, with a phospholipid bilayer structure. Their size and shape can be identified clearly using electron microscopy (Benito-Martin et al., 2013). Exosomes contain several specific membrane transport proteins and fusion proteins, such as RAB, GTPases, annexins, flotillins, and proteins involved in microvesicle body biogenesis. Moreover, lipid membrane formation-related proteins, such as integrin and transmembrane proteins CD9, CD81, CD82, and CD63, play important roles in the formation and structure of exosomes (Azmi et al., 2013). CD63 and β-actin were detected in the extracted exosomes by Western blotting and in the shotgun mass spectra of all three groups, confirming the extraction of exosomes in saliva.

### Association between oral cavity and the occurrence, development, and treatment of IBD

IBD is associated with a systemic immune response, which can lead to inflammation in the gut and damage of other organs. IBD affects not only the intestinal tract but also the mouth. Specific oral lesions include pebble-like lesions, mucogingivitis, and non-specific IBD oral lesions, including aphthous stomatitis and pyostomatitis vegetans. In addition, exosomes from the oral cavity may also affect the intestinal tract, suggesting a mutual role in IBD (Lankarani et al., 2013).

As early as 2009, Rommel et al. (2009) extracted exosomes from colon cancer cells (lim1215 cell). These vesicles promoted tumor progression and metastasis and played an important role in transportation. In this way, we predict IBD colon cells may also secrete exosomes and colon-derived exosomes may be one of the reasons that influence the oral cavity, just as exosomes work in the metastasis in the pancreas cancer which influence the oral cavity (Lau et al., 2013). The animal studies we conduct partly verify our suggestion that there may be an association between exosomes in colon and oral cavity. The origin of exosome in body fluid is mainly from cell secretion. So the exosomes in saliva should be mostly from the oral tissue cells. This is also partly proved in our animal experiments. Animal experiments showed that the expression level of PSMA7 in oral epithelial tissue was similar to that of intestinal inflammation. The expression level of exosomal PSMA7 in human saliva is also similar to the development of intestinal inflammation. The expression of PSMA7 in oral mucosal epithelium is higher in the IBD mice, which is consist with the expression profile of PSMA7 in the colon tissue in the IBD group. Because salivary exosome is mostly secreted by oral tissue, we believe that exosomal PSMA7 in human saliva is a good biomarker for the development of IBD. But because of the characteristics of long transport of exosomes, we cannot rule out some of the exosomes in saliva are also derived from other places, such as the intestinal fluid and cells through *in vivo* circulation. This requires us to carry out deeper research to prove. What’s more, deep studies concerning the mechanism of exosomes between oral cavity and colon need to be done to verify our speculation. From now on, we can only suggest that contents of salivary exosomes can change through the development of IBD.

### Patients with CD and UC exhibited greater diversity and higher levels of salivary exosomal proteins than did healthy controls

Today, proteomics can provide a powerful approach for IBD, especially for the diagnosis and management of IBD. Although a number of biomarkers show high degrees of diagnostic accuracy, proteomic analyses have not replaced endoscopy as the gold-standard diagnostic method. There is a continuing need for a non-invasive method with sufficient specificity and sensitivity to diagnose IBD. A biomarker present in exosomes, which have high relative stability, is one approach for a more accurate diagnosis of IBD (Chan et al., 2016).

In 2011, Choi et al. (2011) investigated exosomes in malignant ascites from colorectal cancer patients. Various cancer cells, including colorectal cancer cells, release exosomes into the surrounding tissue and peripheral circulation, including malignant ascites. In that study, exosomes were extracted from colorectal cancer ascites and subjected to SDS-PAGE and nano-LC-MS/MS 1D analysis; the authors detected a total of 846 proteins in three colorectal cancer patients. The functions of these proteins include tumor development, migration, invasion, growth, immune regulation, and angiogenesis. They also detected a colon-specific surface antigen in colorectal cancer. Proteomics analyses will be helpful to determine the differential features of exosomes in tumor progression and will contribute to the development of new diagnostic tools for colorectal cancer.

We performed a similar process of extracting exosomes from the saliva of patients with IBD and detected exosomal proteins using an advanced shotgun proteomics approach. As a result, we detected 399 and 392 unique proteins in exosomes from patients with UC and CD, respectively. However, there were >2000 exosomal proteins present in both the UC and CD groups, far more than in the healthy controls. Among them, we focused on eight proteins expressed only in the IBD groups. Most of these eight proteins were related to inflammation, proteasomes activity and immune response, pointing to their origin from IBD disease.

### Salivary exosomal PSMA7 is a significant protein biomarker of IBD

Proteasome subunit PSMA7 is a subunit of the 20S proteasome, an intracellular protein. Many studies have shown that the gene associated with inflammation and immunity increases the risk of major depressive disorder and reduces a patient’s response to certain drugs. The proteasome is a basic part that promotes the management function of T cells. One study suggested that proteasomal PSMA7 plays a role in the susceptibility to antidepressants. In that study, in peripheral blood cells and fibroblasts collected from patients, the proteasome was shown to be responsible for the degradation of proteins and to control autoimmune disorders and immune tolerance. These functions are also relevant to disorders of the digestive system (Minelli et al., 2015).

PSMA7 is also considered a novel biomarker of colorectal cancer. Increased expression of PSMA7 is associated with liver metastasis of colon cancer and reduced survival rate of patients, suggesting the potential for proteasome inhibitors in clinical therapeutic applications (Romanuik et al., 2009). Furthermore, it has been shown that PSMA7 plays a role in the progression of colon cancer and may be a unique target for drug treatment. Abnormal PSMA7 activation can significantly regulate colon cancer metastasis. Hu et al. (2009) knocked out PSMA7 in a human colon cancer cell line. Knockdown of PSMA7 in RKO cells inhibited anchorage-independent growth and cell invasion and migration. Additionally, knockdown of PSMA7 can significantly inhibit RKO cell-induced tumors. PSMA7 may be a target site of drugs based on interference with colorectal cancer treatment. In another study that used small interfering RNA to knockdown PSMA7, increased expression of 97 genes was detected in human colorectal cancer cells (HT-29) exhibiting increased apoptosis. This result indicates that downregulated PSMA7 expression can lead to apoptosis of human colorectal cancer cells. PSMA7 is highly expressed in colorectal cancer cells compared with normal colonic tissues. Thus, PSMA7 silencing may cause cancer cells to enter apoptosis (Honma et al., 2009). The mechanism of PSMA7 in colon cancer may also be explained by tumor angiogenesis, which is an important biological process in IBD pathology (Tandle et al., 2009).

In summary, salivary exosomal PSMA7 was found to express much higher in patients with IBD (CD and UC). It may play roles in immune and inflammatory effects in IBD via protection and long-distance transportation of exosomes as it has been reported in other disease in the digestive system. But the exact mechanism between the salivary exosomal PSMA7 and the development of IBD still need to be explored. PSMA7-containing exosomes exist in the saliva in the oral cavity, which may provide a more accurate diagnostic approach for IBD. Other proteins may also be specific to CD and UC patients, which we still need to investigate.

## CONCLUSIONS

In conclusion, the levels of salivary exosomal PSMA7 differed significantly between healthy individuals and patients with UC and CD. Due to their presence at high levels, exosomal proteins produced in IBD may be a sign of the development of the disease, but further study needs to be conducted. We think the salivary exosomal PSMA7 is an ideal biomarker of IBD, and it can also demonstrate the development of IBD, which may release the pain in rectoscope for millions of patients.

## MATERIAL AND METHODS

### Saliva sample collection and preparation

In total, 48 patients were recruited from the People’s Hospital, Peking University, from 2013 to 2015. Among these patients, 37 had UC and the others had CD. We also recruited 10 healthy subjects without IBD as controls. The patients selected in 2013–2015 were newly diagnosed with IBD and were treatment naïve. They were matched in terms of age, sex, and systemic diseases with the control group. And all the patients were examined that none of them has any oral diseases. In 2016, we revisited five of the previous patients who had entered a remission phases. Salivary samples were collected from the recruited individuals. All individuals were asked to rest for 15 min before saliva collection and not to eat or drink after dinner the previous evening or to brush their teeth on the collection day morning. The subjects sat upright in a quiet room and were required to put the tip of their tongue against the sublingual caruncle without straining. Thus, the saliva, which was received in a paper cup for the first 5 min, could run from the mouth, and we collected 5 mL of the spontaneous saliva flow in a 50-mL centrifuge tube. During the collection procedure, patients were asked not to speak. Immediately after collection, the unstimulated whole saliva samples were kept on ice and then centrifuged at 10,000 ×*g*/min for 10 min at 4°C to remove insoluble materials, cells, and debris. The supernatant of the saliva is used for further experiments.

This study was approved by the Peking University Biomedical Ethics Committee. All participants provided written informed consent. The volunteers’ information is provided in Table S1.

### Exosome isolation and transmission electron microscopy

Exosome extraction was performed according to the protocol of the Invitrogen Total Exosome Isolation kit (catalog number: 4484453). We used electron microscopy to identify the shape and size of the substances extracted and to confirm isolation of exosomes. The extracted exosomes were resuspended in 1× PBS. Then, aliquots (5 µL) of the exosome samples were placed on carbon-coated grids (previously treated with plasma cleaner; Ted Pella Inc, CA, USA). The samples were blotted with filter paper after 30 s. Then the samples were stained with 2% uranyl acetate for 1 min. The grids were examined under the FEI T12 electron microscope at 120 kV. The micrographs were taken using a Gatanultra scan 4K × 4K.

### Western blotting of specific exosomal membrane proteins and specific exosomal proteins from patients with IBD

Exosomes were dissociated using Total Exosome RNA and Proteins Isolation kit (Invitrogen) according to the protocol. Western blotting was used to verify specific exosomal membrane proteins and specific proteins from patients with IBD and healthy controls and also, mouse tissue. The primary antibodies (anti-β actin, cat. no. ab6275, anti-CD63, cat. no. ab8219, anti-PSMA7, cat. no. ab133502, and anti-IL6, cat. no. ab7737, all from AbcamInc, Cambridge, Britain) were mixed with milk at a ratio of 1:1000. The secondary anti-IgG fluorescent antibody was mixed with TBST/milk at a ratio of 1:15,000.

### Shotgun mass spectroscopy analysis

Exosomal proteins were extracted from the saliva of patients and healthy controls for shotgun mass spectroscopy analyses. Exosomal proteins were separated by 10% SDS-PAGE, and the sample gel lanes were cut into five or six pieces. Then, the gel pieces were dissolved in methanol and loaded into the LTQ OrbitrapVelos instrument. Protein IDs and original abundance values were obtained from the LC-MS/MS mass spectra.

### Animal studies

Male C57BL/6 mice (6–8 weeks old, weighing 18–22 g) were from the Animal Research Center of Peking University People’s Hospital and were housed in a specifc pathogen-free facility. The experimental protocols were approved by Peking University Biomedical Ethics Committee. Male C57BL/6 mice were treated with 2.5% DSS (dextran sulphate sodium) in drinking water for 10 days to induce colon injury and colitis (Bian et al., 2011). Normal control mice were given normal drinking water. Mice were sacrificed on day 8 by eye bloodletting followed by cervical dislocation. Colons were mechanically isolated, cleaned, and measured in length. Oral mucosa epithelial tissue was also cut out. The oral and colon tissue from the mice were made into tissue lysis by RIPA to do further study.

### Data analysis

We searched the raw data obtained from the LC-MS/MS analysis using the Uniprot website. A gene ontology (GO) analysis was performed using the DAVID website. The data were searched against the human entries in the SwissProt database using the built-in decoy option. The ImageJ software was used to assess differences in Western blotting results among the three groups.

## Electronic supplementary material

Below is the link to the electronic supplementary material.

Supplementary material 1 (PDF 6 kb)

Supplementary material 2 (ZIP 90 kb)
