# Supplementary material for: Salivary exosomal PSMA7: a promising biomarker of inflammatory bowel disease
Source: Protein Cell. 2017 May 18;8(9):686–95. doi: 10.1007/s13238-017-0413-7 (PMC5563283; doi:10.1007/s13238-017-0413-7)
Supplement: Supplementary file 1 — Supplementary material 1 (PDF 6 kb) [file 13238_2017_413_MOESM1_ESM.pdf]

Appendix Table 1. Clinical characterization of the IBD patients

Group	Number	Age(mean)	Gender	Diagnose
Healthy control	10	50.1	6 males and 4 females	Without systematic diseases
UC	37	48.0	18 males and 19 females	Ulcerative colitis
CD	11	45.5	6 males and 5 females	Crohn disease

Appendix Table 1: Samples collected from healthy controls, UC and CD patients have been carefully controlled of the age, genders and systemic diseases. *P* value of age of the three group is 0.7428.
